# Biliary Atresia and Rare Concurrent Cystic Fibrosis Variant: Case Report and Management Considerations

**DOI:** 10.1097/PG9.0000000000000285

**Published:** 2023-01-30

**Authors:** Brandon Ang, Navneet Singh, Mohamed A. Shaban, Jennifer Snider

**Affiliations:** From the * Valley Children’s Healthcare, Madera, CA; † UCSF Fresno Community Medical Regional Center, Fresno, CA.

**Keywords:** cystic fibrosis transmembrane conductance regulator, cystic fibrosis, extrahepatic biliary atresia

## Abstract

It is uncommon for a patient to have 2 different diagnoses contributing to neonatal cholestasis and poor growth. We present a 2-month-old female with extrahepatic biliary atresia status after Kasai procedure at 4 weeks old presenting with persistent neonatal cholestasis. The patient was admitted for intolerance of oral feeds, concern for cholangitis and Kasai failure, and nutritional optimization. She was found to have genetic testing positive for 2 rare cystic fibrosis transmembrane conductance regulator mutations and pancreatic insufficiency consistent with a possible diagnosis of cystic fibrosis–related disease. We discuss the implications and management considerations in a patient with both biliary atresia and cystic fibrosis.

## INTRODUCTION

Neonatal cholestasis should be evaluated expeditiously, as the risk of delaying diagnosis can be life-threatening, specifically in identifying and treating biliary atresia (BA). Evaluation currently includes genetic testing, which often takes time to result. Patients with BA status after Kasai procedure that have persistent neonatal cholestasis should be evaluated for Kasai failure and disease progression. We report a patient with BA status after Kasai procedure who presented with persistent cholestasis and poor growth incidentally found with 2 rare cystic fibrosis (CF) transmembrane conductance regulator (CFTR) genetic mutations and pancreatic insufficiency, consistent with a concurrent mild presentation of CF-related disease.

## CASE REPORT

A 2-month-old female with a history of BA status after Kasai procedure at 4 weeks old presented with nonbloody, nonbilious emesis, and rising liver enzyme tests (Table 1). Differential diagnosis included ascending cholangitis versus Kasai failure. She was afebrile with stable normal vital signs. Physical examination revealed a malnourished (underweight) baby with jaundice, scleral icterus, and a distended, soft abdomen with active bowel sounds and no hepatosplenomegaly. Stools were slightly yellow pigmented. Medications included ursodiol, ADEKs vitamins, and trimethoprim-sulfamethoxazole for cholangitis prophylaxis.

**TABLE 1.** Patient’s liver enzyme and bilirubin levels after Kasai procedure

Postoperative day	Day 5	Day 15	Day 23	Day 25 (day of admission)
ALP (U/L)	330	397	434	446
AST (U/L)	116	154	158	192
ALT (U/L)	109	75	94	106
Direct bilirubin (mg/dL)	4.8	4.9	7.2	6.6
Total bilirubin (mg/dL)	5.7	6.7	8.9	9.3
GGT (U/L)	1364		2436	2696

ALP = alkaline phosphatase; ALT = alanine transaminase; AST = aspartate transaminase; GGT = gamma-glutamyl transferase.

From the history, she initially presented at 1-week old with direct hyperbilirubinemia and acholic stools. Abdominal ultrasound showed a contracted gallbladder. Evaluation for infectious causes was negative, and genetic cholestasis panel was sent. At 1-month old, intraoperative cholangiogram revealed no obvious gallbladder lumen. The distal aspect of the gallbladder fundus was excised to find a small, blind-ending lumen. The gallbladder connected to scarred extrahepatic biliary ducts with no lumen. Cholangiogram was not possible. She was diagnosed with BA and underwent Kasai procedure.

On this admission, the patient was treated for suspected cholangitis. Abdominal ultrasound showed a hypoechogenicity in the left hepatic lobe concerning for an abscess but without ring enhancement and was diagnosed as a biloma. An upper gastrointestinal series ruled out volvulus. computerized tomography abdomen and pelvis showed no acute findings except expected post-surgical changes. The patient was also diagnosed with failure to thrive with lack of weight gain despite high caloric intake on high medium-triglyceride formula either orally or via nasogastric feedings.

Incidentally, her genetic cholestasis panel resulted with 2 variants in the CFTR gene associated with CF. Sweat chloride test was normal (22 mmol/L), but fecal elastase testing revealed severe pancreatic insufficiency. Parents were also tested, revealing the mother had the same variants. Weight gain improved after starting pancreatic enzyme replacement, but liver enzyme levels and liver functions including prothrombin time and total and direct bilirubin levels worsened (Table 2). She was transferred to a transplant center for possible liver transplant evaluation and further management.

**TABLE 2.** Patient’s liver enzyme levels and function tests while hospitalized

	Day 1	Day 3	Day 7	Day 10	Day 17
ALP (U/L)	446	382	381	376	479
AST (U/L)	192	131	140	132	231
ALT (U/L)	106	93	107	102	150
Direct bilirubin (mg/dL)	6.6	6.4	6.2	5.7	5.1
Total bilirubin (mg/dL)	9.3	7.9	7.8	7.2	6.6
Ammonia (μmol/L)	60	78	49	48	42
GGT (U/L)	2696	2382	2338	2336	2981
PT	13.2	15.1	17.4	-	13.7
INR	1.0	1.1	1.4	-	1.0

Stool pancreatic elastase testing resulted low on day 10, and enzyme replacement was added on day 14.

ALP = alkaline phosphatase; ALT = alanine transaminase; AST = aspartate transaminase; GGT = gamma-glutamyl transferase.

## DISCUSSION

**FIGURE 1.**
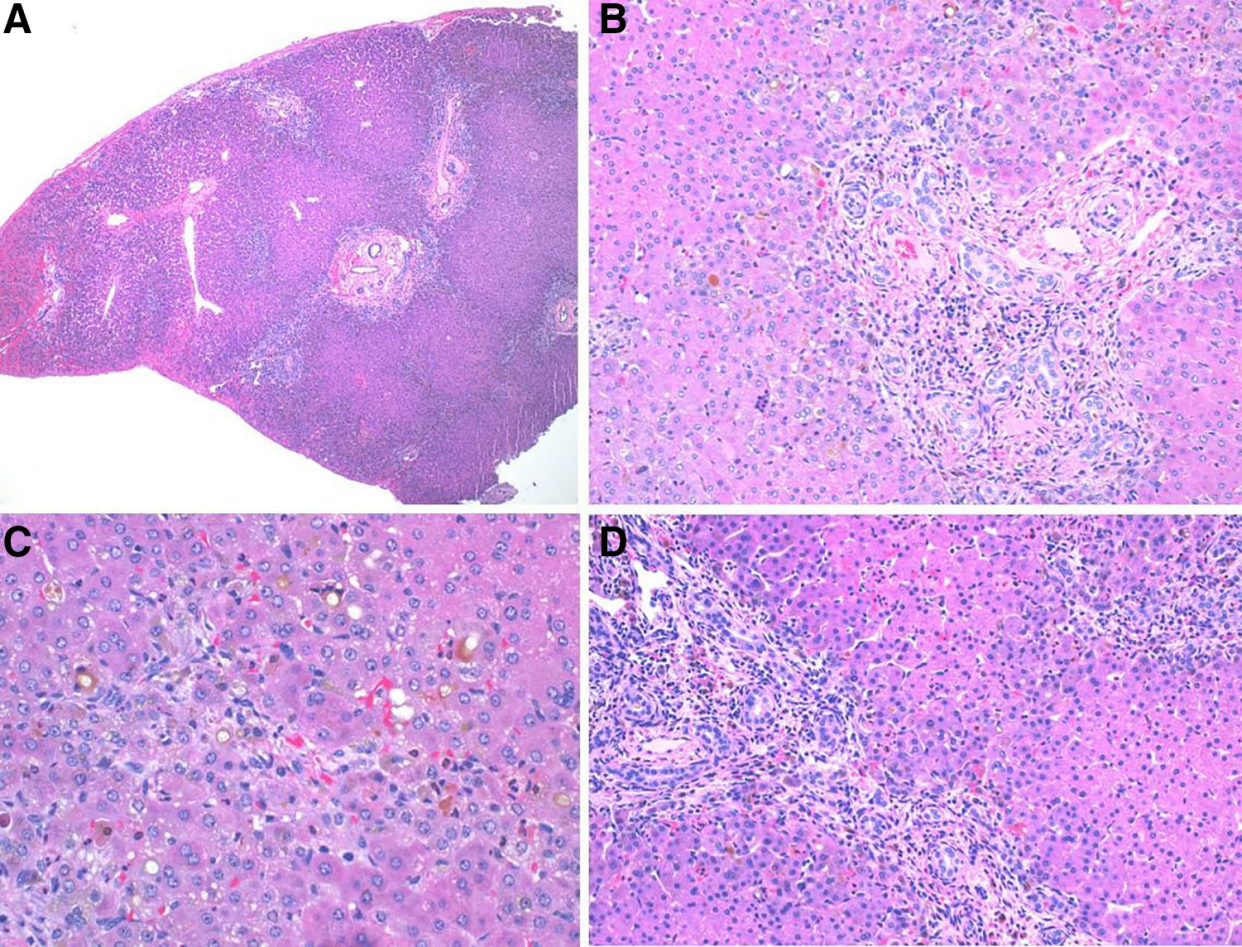
Liver biopsy from day of Kasai with H&E stain under low, medium, and high power showing fibrous portal expansion and ductular reaction consistent with biliary obstructive pattern of injury, but no specific finding to distinguish biliary atresia from cystic fibrosis.

BA with concurrent CF has not been reported previously, but CF can present as neonatal cholestasis and has been mistaken for BA (1-3). Cholestasis in CF is due to the presence of defective CFTR proteins in the biliary ducts and has been documented in several patients to present solely with conjugated hyperbilirubinemia (2), but the incidence is as rare as 0.6% (3). Although cases of CF cholangiopathy have been mistaken for BA (4,5), we are confident that our patient was correctly diagnosed with BA given intraoperative findings of an atretic extrahepatic common bile duct. Findings on liver histology show intrahepatic bile duct reaction without paucity, overall consistent with biliary obstruction seen in BA (Fig. 1). She underwent appropriate Kasai, but the patient’s presentation may have included CFTR mutation-related effects not considered until the genetic panel resulted. CF-related cholestasis and pancreatic insufficiency could have played a role in the patient’s continued cholestasis and failure to thrive, ultimately hastening potential Kasai failure, worsening prognosis, and necessitating early liver transplant evaluation.

The patient’s specific genetic findings, heterozygous for CFTR c.935_937del.p (aka. Phe312del) and heterozygous for CFTR c.1210-7_1210-6delTT (aka. 5T), are rare variants of the CFTR gene. The Phe312del variant is considered pathogenic, and there has been 1 reported case in the literature, and 26 patients in the CFTR2 database with 46% reportedly being pancreatic insufficient (6,7). The 5T variant is considered CF modifying, as it contributes to CF disease in the presence of another pathogenic variant (8). The combination of Phe312del and 5T is not reported in the CFTR2 database (6). Neither parent had a family history of CF, the patient had a normal newborn screen, and she did not have risk factors classically associated with CF (male gender, meconium ileus, early positive sweat chloride test) (9). The patient likely directly inherited the mutations from the mother as they had the same mutations. Significant clinical variability in phenotypes is associated with heterozygous CFTR mutations, possibly explaining the lack of reported symptoms by the mother. Heterozygous carriers of CFTR mutations may not develop classic CF symptoms until later in life but do carry increased risk of pancreatic manifestations, which could explain the patient’s nonclassic presentation (10).

While the patient’s sweat chloride test was negative, false negatives are possible while malnourished and do not exclude active CF disease (11). The patient’s persistent neonatal cholestasis and failure to thrive could also be due solely to the natural course of BA disease progression and Kasai failure, however failure to thrive has also been reported in CF-associated neonatal cholestasis (2). Additionally, the patient’s synthetic liver function remained stable, but sustained direct hyperbilirubinemia and elevated gamma-glutamyl transferase may suggest the persistent cholestasis was less likely driven by Kasai failure (Table 2). Pancreatic insufficiency from CF-related disease as evidenced by low fecal elastase and improved weight gain after starting pancreatic enzyme replacement supports an additional pathogenic process other than Kasai failure in the patient.

In the most current 2017 North American Society For Pediatric Gastroenterology, Hepatology & Nutrition guidelines for evaluation of the cholestatic infant, genetic testing including cholestasis gene panels and genetic CFTR testing are recommended as second tier investigations (12). As future cases of CF from rare genetic variants/modifiers are reported, we can better characterize the extent of disease related to these variants. By addressing CF-related failure to thrive from malabsorption and pancreatic insufficiency early, overall patient prognosis could be improved through growth optimization. Evaluation for and consideration of CF-related disease in patients with concurrent BA and CF could affect decision-making for liver transplantation timing in the setting of Kasai failure or BA disease progression.

## ACKNOWLEDGMENTS

Informed consent was obtained from the parents for publication of the details of this case.
